# Novel Biomarkers of Gastrointestinal Cancer

**DOI:** 10.3390/cancers13071501

**Published:** 2021-03-25

**Authors:** Takaya Shimura

**Affiliations:** Department of Gastroenterology and Metabolism, Graduate School of Medical Sciences, Nagoya City University, Nagoya 467-8601, Japan; tshimura@med.nagoya-cu.ac.jp; Tel.: +81-52-853-8211; Fax: +81-52-852-0952

Gastrointestinal (GI) cancer is a major cause of morbidity and mortality worldwide. Among the top seven malignancies with worst mortality, GI cancer consists of five cancers: colorectal cancer (CRC), liver cancer, gastric cancer (GC), esophageal cancer, and pancreatic cancer, which are the second, third, fourth, sixth, and seventh leading causes of cancer death worldwide, respectively [1]. To improve the prognosis of GI cancers, scientific and technical development is required for both diagnostic and therapeutic strategies.

## 1. Diagnostic Biomarker

As for diagnosis, needless to say, early detection is the first priority to prevent cancer death. The gold standard diagnostic tool is objective examination using an imaging instrument, including endoscopy and computed tomography, and the final diagnosis is established with pathologic diagnosis using biopsy samples obtained through endoscopy, ultrasonography, and endoscopic ultrasonography. Clinical and pathological information is definitely needed before the initiation of treatment because they could clarify the specific type and extent of disease. However, these imaging examinations have not been recommended as screening tests for healthy individuals due to their invasiveness and high cost. Hence, the discovery of novel non-invasive biomarkers is needed in detecting GI cancers. In particular, non-invasive samples, such as blood, urine, feces, and saliva, are promising diagnostic biomarker samples for screening.

Stool-based tests, including guaiac fecal occult blood test (gFOBT) and fecal immunochemical test for hemoglobin (FIT), for CRC have been some of the most successful screening tests for GI cancers. Although the gFOBT is the only non-invasive method that demonstrated a reduction in CRC mortality [2], the FIT recently gained popularity because the FIT has a higher sensitivity for CRC and adenoma than gFOBT [3] due to its specificity for human globin. However, since both gFOBT and FIT aim to detect only blood contamination in feces, which is not cancer-specific, sensitivity for early-stage cancer and advanced adenoma is quite low. Moreover, in handling stool samples, stool-based tests are challenging for both patients and investigators, and the quality of sample collection by patients may affect the results.

Fortunately, recent technical and mechanical developments have enabled the detection of slight differences in factors that are modified in physical condition, which might contribute to novel biomarker discovery for GI cancers. Analytical targets include a wide variety of factors, including DNA mutation, DNA methylation, miRNA, protein, and metabolites [4]. Moreover, many types of body fluids are target samples. Although blood is the most popular biomarker sample, other samples, including urine, saliva, and sweat are also attractive tools because of their non-invasiveness.

Diagnostic biomarkers are mostly developed in the field of CRC, including blood- and stool-based biomarkers. As expected, blood-based test is more preferred than a stool-based test as an alternative noninvasive test, and Epi proColon^®^ 2.0 CE is an FDA-approved blood test for CRC screening, which detects methylated Septin9 DNA [5]. However, this blood-based test has not been recommended as a screening test for CRC due to its low sensitivity and limited data [6]. Presently, many researchers are trying to explore reliable blood-based biomarkers for detecting GI cancers. Among these, cell-free DNA, miRNA, and proteomics approaches are the most common targets. However, these novel biomarkers are still under study, and we expect future clinical application with reliable validation.

In contrast, there are far fewer studies on urinary biomarkers than on blood-based biomarkers for GI cancers. We and another group previously reported the usefulness of urinary protein and miRNA biomarkers in detecting GI cancers [4,7,8,9,10]. As urine is a completely non-invasive sample, urinary biomarker enables screening tests at home. The advantages of urinary biomarkers with easy access and low cost might improve screening compliance, which may result in a reduction in GI cancer mortalities.

## 2. Treatment Biomarker

In terms of treatment biomarkers, since patients have already been diagnosed with some types of cancers, invasive sampling from tissue, bile, and pancreatic juice is accepted, which can be generally obtained through close examination. Indeed, some tissue-based biomarkers have already been applied to clinical practices of GC and CRC. Positive expression of human epidermal growth factor receptor 2 (HER2) in GC tissues is a predictive biomarker for anti-HER2 antibody, trastuzumab, in advanced GC [11] as well as HER2-positive breast cancer.

Tumor *RAS* mutation representing mutation in exons 2, 3, and 4 of *KRAS* and *NRAS* is a negative predictive biomarker for anti-EGFR antibody therapy against metastatic CRC [12]. Moreover, BRAF inhibitor has been applied for metastatic CRC with *BRAF* V600E mutation in tumor tissues [13]. Likewise, immune checkpoint inhibitors have been applied for metastatic CRC with high microsatellite instability or mismatch-repair deficiency [14,15]. Precision medicine based on these predictive biomarkers contributes to not only better prognosis and safety but also cost reduction by avoiding unnecessary treatment.

Moreover, liquid biopsy detecting circulating cell-free DNA has been recently applied as an alternative test to the tissue-based *RAS* mutation test, which showed a high concordance rate between plasma and tissue-based results [16]. Liquid biopsy, which comprises body fluid-based biomarkers, has a huge benefit compared to tissue-based biopsy because it easily enables repeated sampling depending on the systemic physical situation. This benefit is especially useful in monitoring during a specific therapy and follow-up observation after tumor resection. Since malignant tumors consist of heterogenous cells, the characteristic of dominant cancer cells might be dynamically changed in a time-dependent manner. Additionally, the microenvironment surrounding tumors also changes dynamically. Since liquid biopsy through circulating body fluid might systemically capture these dynamic changes, it can be applied for monitoring biomarker beyond treatment biomarker. In fact, a Signatera^TM^ test detecting custom-built plasma cell-free DNAs could predict relapse after surgical resection of stage I–III CRC with high sensitivity [17]. Liquid biopsy is presently in the initial phase, and future development is expected in many fields of GI cancers for predicting efficacy, adverse events, and recurrence.
